# GC-MS Profile of Hua-Feng-Dan and RNA-Seq Analysis of Induced Adaptive Responses in the Liver

**DOI:** 10.3389/fphar.2022.730318

**Published:** 2022-03-08

**Authors:** Jia-Jia Liu, Yan Liang, Ya Zhang, Rui-Xia Wu, Ying-Lian Song, Feng Zhang, Jing-Shan Shi, Jie Liu, Shang-Fu Xu, Zhang Wang

**Affiliations:** ^1^ Key Laboratory of Basic Pharmacology of Ministry of Education and Joint International Research Laboratory of Ethnocentric of Ministry of Education, Zunyi Medical University, Zunyi, China; ^2^ College of Pharmacy, Chengdu University of Traditional Chinese Medicine, Chengdu, China; ^3^ College of Ethnomedicine, Chengdu University of Traditional Chinese Medicine, Chengdu, China

**Keywords:** Hua-Feng-Dan, RNA-seq, GC-MS, bioinformatics, adaptation, GEO database

## Abstract

**Background:** Hua-Feng-Dan is a patent Chinese medicine for stroke recovery and various diseases. This study used GC-MS to profile its ingredients and RNA-Seq to analyze the induced adaptive response in the liver.

**Methods:** Hua-Feng-Dan was subjected to steam distillation and solvent extraction, followed by GC-MS analysis. Mice were orally administered Hua-Feng-Dan and its “Guide drug” Yaomu for 7 days. Liver pathology was examined, and total RNA isolated for RNA-Seq, followed by bioinformatic analysis and quantitative real-time PCR (qPCR).

**Results:** Forty-four volatile and fifty liposoluble components in Hua-Feng-Dan were profiled and analyzed by the NIST library and their concentrations quantified. The major components (>1%) in volatile (5) and liposoluble (10) were highlighted. Hua-Feng-Dan and Yaomu at hepatoprotective doses did not produce liver toxicity as evidenced by histopathology and serum enzyme activities. GO Enrichment revealed that Hua-Feng-Dan affected lipid homeostasis, protein folding, and cell adhesion. KEGG showed activated cholesterol metabolism, bile secretion, and PPAR signaling pathways. Differentially expressed genes (DEGs) were identified by DESeq2 with *p* < 0.05 compared to controls. Hua-Feng-Dan produced more DEGs than Yaomu. qPCR on selected genes largely verified RNA-Seq results. Ingenuity Pathways Analysis of the upstream regulator revealed activation of MAPK and adaptive responses by Hua-Feng-Dan, and Yaomu was less effective. Hua-Feng-Dan-induced DEGs were highly correlated with the Gene Expression Omnibus database of chemical-induced adaptive transcriptome changes in the liver.

**Conclusion:** GC-MS primarily profiled volatile and liposoluble components in Hua-Feng-Dan. Hua-Feng-Dan at the hepatoprotective dose did not produce liver pathological changes but induced metabolic and signaling pathway activations. The effects of Hua-Feng-Dan on liver transcriptome changes point toward induced adaptive responses to program the liver to produce hepatoprotective effects.

## Highlights

• GC-MS profiled 44 volatile and 50 liposoluble components in Hua-Feng-Dan (HFD).
• RNA-Seq analyzed HFD-induced adaptive transcriptome changes in the liver.
• HFD produced more differentially expressed genes than its Guide drug Yaomu.
• HFD activated MAPK and induced adaptive responses to “program the liver.”
• HFD Effects were correlated with GEO database of chemical-induced adaptive changes.

## Introduction

Hua-Feng-Dan has over 300 years of history in the treatment of stroke and various diseases in China and is still used today alone or in combination with other medications (Liu et al., 2018). Hua-Feng-Dan contains cinnabar (HgS) and realgar (As_4_S_4_), and our initial research focused on the safety evaluation of Hua-Feng-Dan following acute (Yan et al., 2011), subacute (Peng et al., 2012; Zhu et al., 2014), and subchronic (Chen et al., 2020; Hu et al., 2020) administrations and demonstrated that the toxicity of Hua-Feng-Dan is different from that of environmental mercury (Hg) and arsenic (As) compounds (Liu et al., 2018). In addition to cinnabar and realgar, Hua-Feng-Dan also contains herbs (*Gastrodia elata* Blume.; *Nepeta tenuifolia* Benth; *Croton tiglium* L.; *Atractylodes lancea* (Thunb.) DC.; *Typhonium giganteum* Engl.; *Blumea balsamifera* (L.) DC.; *Acorus tatarinowii* Schott; *Perilla frutescens* (L.) Britt.), animal-based products (*Moschus berezovskii* Flerov (Shexiang); *Buthus martensii* Karsch (Quanxie), *Bombyx mori* Linnaeus (Jiangcan), and other minerals (Borax; Borneolum Syntheticum) (Liu et al., 2018). Network pharmacology has found that β-sitosterol, luteolin, baicalein, and wogoni are potential active ingredients (Yang et al., 2020), but experimental verification is required. We have demonstrated the protective effects of Hua-Feng-Dan against LPS plus MPTP-induced Parkinson’s disease (PD) mouse model (Hu et al., 2020), and LPS plus rotenone-induced PD rat model (Chen et al., 2020), and identified that cinnabar and realgar are two active ingredients in the recipe both in LPS-induced neuroinflammation in rat midbrain neuron–glia cocultures (Zhang et al., 2012a) and in LPS plus rotenone-induced dopaminergic neuron loss in rats (Chen et al., 2020). We have also found that Hua-Feng-Dan had modulatory effects on gut microbiota in PD mice (Hu et al., 2020) and PD rats (Chen et al., 2020).

Yaomu is considered as the “Guide Drug” in Hua-Feng-Dan. “Guide drug” is an important theory in Chinese medicines as exemplified by licorice (also called Gan-Cao, *Glycyrrhiza uralensis* Fisch). Licorice as a “Guide Drug” is essential to enhancing efficacy and reducing toxicity when combined with other herbal medicine preparations (Wang et al., 2013; Jiang et al., 2020). Yaomu in Hua-Feng-Dan consists of 7 components (*Typhonium giganteum* Engl; *Pinellia ternata* (Thunb.) Breit; *Arisaema heterophyllum* Blume; *Aconitum* carmichaelii Debx; *Curcuma wenyujin* Y. H. Chen et C. Ling; Medicated Leaven (Shen-Qu); and cow bile water) and is subjected to fermentation for 3 months. Fermentation is an important processing technology in traditional medicines under appropriate temperature, humidity, and microorganisms to reduce toxicity, enhance efficacy, and generate active inter-metabolites (Li et al., 2020). Fermented Yaomu could reduce the content and toxicity of aconitine, mesaconitine, and hypaconitine (Cao et al., 2019), with increases in monoester alkaloids and decreases in diester alkaloids (Ma et al., 2020). The contents of 7 nucleotides also changed with fermentation time (Cao et al., 2021). In a serum pharmacochemistry study of Hua-Feng-Dan and Yaomu by ultrahigh-performance liquid chromatography-time-of flight mass spectrometry, 8 drug-derived components (6 from Yaomu) were found in serum under negative ion mode, while 9 drug-induced components (6 from Yaomu) were found in serum under positive ion mode (Xiang et al., 2016).

However, little is known about the chemical ingredients in Hua-Feng-Dan. The gas chromatography–mass spectrometry (GC-MS) was successfully applied in our recent publications to identify volatile and liposoluble components in Qishiwei Zhenzhu pills (Xu et al., 2020) and chemical constituents in *Prunus mira* Koehne (Sun et al., 2018; Zhou et al., 2020). The same strategy was used in this study to primarily profile volatile and liposoluble components in Hua-Feng-Dan.

The liver is the main organ of the phase I and phase II drug metabolism and plays a leading role in the elimination of oral drugs (Klaassen, 2014). The liver is also an important organ of detoxification in response to xenobiotics. To “program the liver” is an important concept in that xenobiotics at appropriate doses could evoke adaptive responses to induce a number of molecular events to protect against toxic stimuli (Klaassen, 2014). For example, oleanolic acid, a triterpenoid from many herbs/fruits, could reprogram the liver to protect against a variety of hepatotoxicants at the low doses but is hepatotoxic at higher doses (Liu et al., 2019). We have recently found that Hua-Feng-Dan protected against CCl_4_-induced liver injury (manuscript in preparation). Thus, learning to program the liver would help us understand the hepatoprotective effects of Hua-Feng-Dan.

We have recently used RNA-Seq to reveal adaptive mechanisms for *Dendrobium nobile* alkaloids to protect against CCl_4_-induced liver injury (Zhang et al., 2021). This study used the same strategy to profile the liver transcriptome after Hua-Feng-Dan and Yaomu treatment. After quality evaluation of RNA-Seq data, differentially expressed genes (DEGs) were identified with the DESeq2 method and qPCR verified selected genes. A comprehensive bioinformatics including two-dimensional clustering, Gene Ontology (GO) and Kyoto Encyclopedia of Genes and Genomes (KEGG) enrichment analysis, Ingenuity Pathways Analysis (IPA), and Illumina BaseSpace Correlation Engine (BSCE) analysis was used to analyze DEGs and to mine induced adaptive responses.

## 2 Materials and Methods

### 2.1 Materials

Hua-Feng-Dan (HFD) and its Guide Drug Yaomu (YM) were provided by Hua-Feng-Dan Pharmaceutical Co. (Guizhou, China). All other chemicals were of reagent grade.

### 2.2 Gas Chromatography-Mass Analysis of Volatile Components in Hua-Feng-Dan

An Agilent 5975C GC-MS system (Agilent Technologies, Lexington, MA, United States) with a HP-5MS chromatography column (30 m ×0.25 mm × 0.25 μm) was used for chromatographic analysis as described previously (Xu et al., 2020).

Approximately 5 g of Hua-Feng-Dan powder was mixed with 200 ml water and extracted for 2 h with a volatile oil extractor. The resultant liquid was dissolved in 2 ml of petroleum ether, and the volume was fixed in a 5-ml volumetric flask. GC-MS steam distillation: the program temperature started at 40°C and maintained for 3 min, then increased to 52°C at a rate of 1°C/min, and continued to increase to 160°C at a rate of 20°C/min, and finally increased to 280°C at a rate of 5°C/min, and maintained for 3 min. The carrier gas was nitrogen (99.999%), the volume flow rate was 1 ml/min, the shunt ratio was 10:1, and the injection volume was 1 μl. The solvent was delayed by 3.5 min. The ion source of MS was electron ionization with 70 eV at 230°C. The specific conditions of MS analysis were as follows: interface temperature, 280°C; quadruple temperature, 150°C; full-scan mode, m/z 35–550 amu.

### 2.3 GC-MS Analysis of Liposoluble Components in Hua-Feng-Dan

Approximately 2 g of Hua-Feng-Dan powder was mixed with 20 ml of n-hexane and sonicated for 30 min, extracted twice, and filtered. The filtrate was evaporated using a rotary evaporator (Shanghai, China) and redissolved with 3 ml of n-hexane. The solution was centrifuged (3,000 rpm/min, 15 min), and the supernatant was transferred into a 5-ml volumetric bottle and filtered through a 0.45-μm membrane. GC-MS solvent extraction: the program temperature started at 60°C, and then increased to 130°C at a rate of 20°C/min, finally increased to 280°C at a rate of 10°C/min, and maintained for 15 min. The temperature of the vaporization chamber was 280°C. The carrier gas was nitrogen (99.999%), the volume flow rate was 1 ml/min, the shunt ratio was 10:1, and the injection volume was 1 μl. The solvent was delayed by 3.0 min. The MS condition is the same as in *Gas Chromatography–Mass Analysis of Volatile Components in Hua-Feng-Dan*.

### 2.4 Animals and Drug Treatment

Adult male C57BL/6J mice (20–22 g) were obtained from Beijing Biotechnology Co., Ltd. (certificate No. SCXK, 2101-0011). The experimental animals were kept in the SPF animal room of the Key Laboratory of Basic Pharmacology of Ministry of Education, Zunyi Medical University, at a temperature of 20 ± 2°C, with lighting from 8:00 in the morning to 8:00 in the evening and free access to food and water. The experimental protocol followed the Chinese Animal Protection and Welfare Guidelines and was approved by the Animal Ethics Committee of Zunyi Medical University (2015-07).

After acclimatization for 1 week, mice were randomly divided into four groups: Control group (*n* = 5), YM-0.1 (*n* = 5, Yaomu 0.1 g/kg), YM-0.3 (*n* = 7, Yaomu 0.3 g/kg), and HFD (*n* = 5, Hua-Feng-Dan 1.2 g/kg). All drugs were suspended in 0.3% sodium carboxymethyl cellulose. Mice received oral gavage daily for 7 consecutive days; the dose of Hua-Feng-Dan selection was based on recent publications (Chen et al., 2020; Hu et al., 2020), and Yaomu was estimated from the recipe. Twenty-four hours after the last administration, mice were anesthetized with sodium pentobarbital (65 mg/kg, ip) and blood, and livers were collected.

### 2.5 Histopathology

A portion of livers from the same site was cut and fixed with 10% formaldehyde, dehydrated with ethanol gradients, and embedded in paraffin. Liver tissue was cut into 4-µm slices with a tissue slicer (model: RM2245), and sections were deparaffinized with xylene, hydrated with gradient ethanol, stained with conventional HE, and observed under upright optical microscope (Olympus, Tokyo, Japan).

### 2.6 Biochemical Analysis

Blood was collected and left standing at room temperature for 2 h and centrifuged at 3,500 rpm for 10 min to separate the serum. The alanine transaminase (ALT) and aspartate aminotransferase (AST) test kits were used to detect the activities of ALT and AST in serum (Nanjing Jiancheng Institute of Bioengineering, Nanjing, China).

### 2.7 RNA Extraction and RNA-Seq

Total RNA was extracted from mouse liver tissues using RNAiso Plus (TAKARA, Dalian, China). The concentration of total RNA is measured with ND-2000 NanoDrop, with A260/A280 > 1.8.

The EASY RNA-Seq method was used to complete the construction of the sample library, and after quality control testing, the Illumina platform was used for sequencing by Chongqing Weilang Biotechnology Co., Ltd. (Chongqing, China), as described in our recent publication (Zhang et al., 2021). Briefly, RNA was subjected to reverse transcription reaction with an oligo (dT) primer to generate the first-strand cDNA; through the joint reaction of RNase H enzyme, DNA polymerase, and T4 ligase, double-strand cDNA was generated. The Agencourt AMPure XP Beads were used to purify cDNA by magnetic separation. After the double-stranded cDNA was fragmented by Tn5 enzyme, the samples were then barcoded following the protocol of TruePrep DNA Library Prep Kit with P5/P7 adapter primers (Vazyme, Nanjing, China, cat. TD503). Sequencing primers were at both ends, and PCR was amplified using the VAHTS™ DNA Clean Beads Kit (Vazyme, cat. N411-02). The Illumina HiSeq platform with a 150-bp double-ended model was used for sequencing analysis.

### 2.8 Bioinformatics

RNA-Seq-generated fragments per kilobase of exon per million fragments mapped (FPKM) were transformed to raw gene counts for quality control analysis with all parameters performed by the sequencing company (Chongqing Weilang Biotechnology Co., Ltd., China) (Zhang et al., 2021). DEGs were identified by the DESeq2 method, and the criteria were set at *p* < 0.05 compared to Controls.

#### 2.8.1 Gene ontology and KEGG Pathway Enrichment Analysis

DEGs were analyzed by GO enrichment, and the hypergeometric distribution was used to test the significantly enriched GO entries (Zhang et al., 2021). ClusterProfiler software (Yu et al., 2012) was used to analyze KEGG pathway enrichment of DEGs among groups. The bubble charts were used to visualize the top functional pathways in GO and KEGG enrichments. The size of the dot in the figure represents the number of differential genes that can be annotated in the functional pathway, and the shade of the color represents the significant degree of enrichment of the functional pathway. Rich factor is a score used to evaluate the degree of enrichment of the functional pathway (Zhang et al., 2021).

#### 2.8.2 Two-Dimensional Clustering

The Gene Cluster version 3.0 (https://cluster2.software.informer.com/3.0/) was used to generate hierarchical complete linkage of DEGs, and the generated.cdt file was then uploaded into TreeView (https://treeview.software.informer.com/1.6/) to generate the heatmap for visualization.

#### 2.8.3 Ingenuity Pathway Analysis

The Ingenuity Pathway Analysis (IPA) server (Qiagen, Redwood City, CA) was used to perform upstream regulator analysis. DEGs (*p* < 0.05) from HFD, YM-0.1, and YM-0.3 treatments vs. control, respectively, were subjected to IPA Core Analysis, followed by comparative analysis to compare the Z-score of the changes among treatment groups.

#### 2.8.4 BaseSpace Correlation Engine analysis

BaseSpace Correlation Engine (BSCE, Illumina, CA) is the RNA sequencing and microarray database curated over 23,000 scientific studies to get data-driven answers for genes, experiments, drugs, and phenotypes for the research. DEGs from various treatment groups vs. Controls were uploaded to BSCE and compared with all biosets in the database using the Running Fisher test. This method provides an assessment of the statistical significance of the correlation of the overlapping genes between DEGs and biosets curated in BSCE, with a summary p-value. The results were exported, and each p-value was converted to a -log (p-value). Biosets with -log (p-value) +4 or −4 were considered a significant correlation (Corton et al., 2019). A column in the gene expression spreadsheet was populated with the -log (p-value)s for each bioset. Biosets that were positively correlated with the DEGs were predicted to produce similar effects, either directly or indirectly (Zhang et al., 2021).

### 2.9 Quantitative Real-Time PCR

The total RNA was reverse transcribed with the PrimeScript RT kit (TAKARA, Dalian, China). The iTaq™ Universal SYBR Green PCR Supermix (Bio-Rad, Hercules, CA, United States) was used for real-time qPCR analysis. The reaction parameters were 95°C 3 min, 95°C 10 s, 60°C 45 s, 40 cycles in total. The Ct values were normalized to β-actin of the same sample and calculated by the 2^−△△Ct^ method and expressed as percentage of Control. The primers were designed by Primer3 (3-4-Supplementary Table S1-primer) and synthesized by Shanghai Sangon Biotech as described (Hu et al., 2020; Zhang et al., 2021).

### 2.10 Statistical analysis

Statistical analysis was carried out using SigmaPlot (v14) software. All values are expressed as mean ± SEM. Data were subjected to the normality test (Shapiro–Wilk) and one-way ANOVA (Kruskal–Wallis), followed by Dunn’s multiple-range tests. The significance level was set at *p* < 0.05 in all cases. DEGs from RNA-Seq were analyzed *via* the DESeq2 method (*p* < 0.05), followed by bioinformatics analysis. A Running Fisher test was used to generate –log (p-value)s for GEO database comparison (Corton et al., 2019; Jackson et al., 2020).

## 3 Results

### 3.1 Test Results of Volatile Oil and Liposoluble Components in Hua-Feng-Dan

**FIGURE 1:**
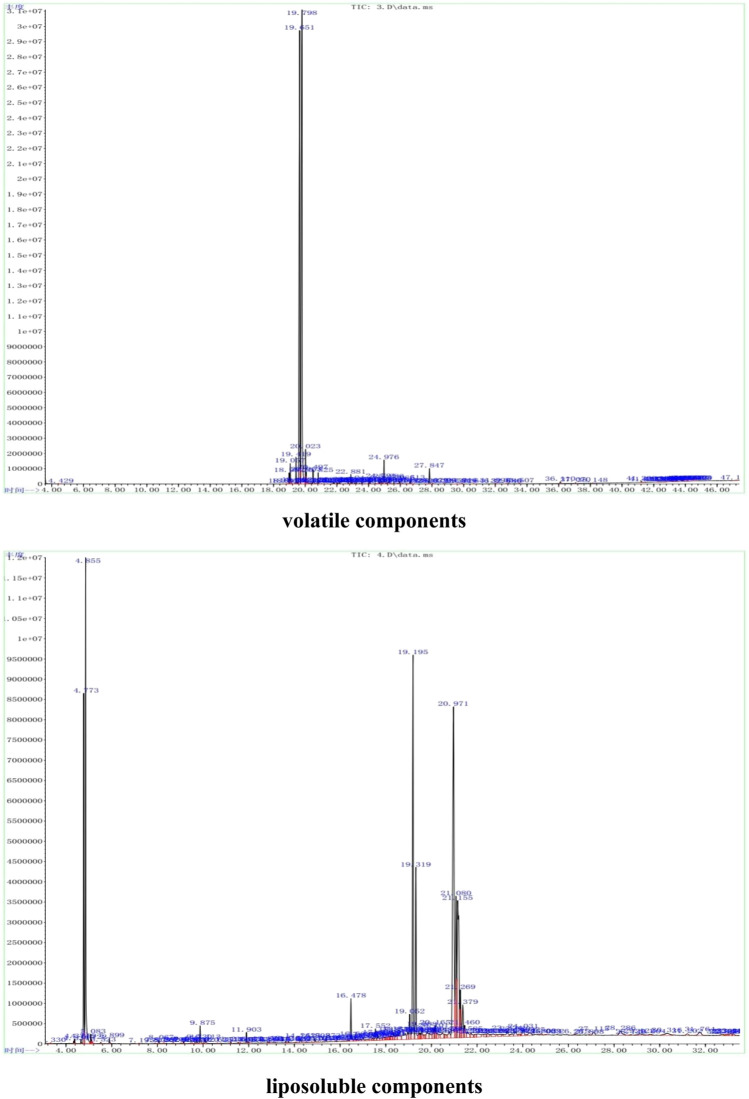
Representative GC-MS analysis of volatile (*via* steam distillation) and liposobuble (*via* solvent extract) components using the strategy as in Xu et al. (2020).

The representative total ion current (TIC) chromatograms of Hua-Feng-Dan are shown in Figure 1. The volatile and liposoluble components in Hua-Feng-Dan were primarily identified and analyzed by searching the National Institute of Standards and Technology (NIST) library as described previously (Xu et al., 2020), and the chromatographic peaks were analyzed and confirmed.

The volatile components in the powder were extracted using steam distillation. A total of 44 compounds were primarily identified, which were divided into ten categories using functional group classification: alcohols (11, relative percent 89.94%), ketones (5, 2.22%), olefins (7, 0.80%), acetate (2, 0.43%), esters (6, 0.28%), aldehydes (1, 0.22%), amine (1, 0.17%). phenols (2, 0.11%), ethers (1, 0.11%), and benzene (1, 0.03%). Among them, alcohols were the most abundant, followed by ketones. Table 1 lists the 44 volatile components in Hua-Feng-Dan. The alcohols include endo-borneol, isoborneol, agarospirol, and (-)-spathulenol; the ketones include (+)-2-Bornanone, muscone, fenchone, 5-methyl-2-(1-methylethylidene)-Cyclohexanone, and 6,10,14-trimethyl-2-pentadecanone. The olefins mainly include caryophyllene, hexadecane, and caryophyllene oxide. The acetate includes bicyclo[2.2.1]heptan-2-ol, 1,7,7-trimethyl-, 2-acetate, cis-hex-3-enyl-phthalic acid. The esters mainly include isobornyl propionate and bornyl isovalerate. The aldehyde includes [1,1′-biphenyl]-4-carboxaldehyde. The amine includes (Z)-9-Octadecenamide. The phenols include 2,2′-methylenebis-(4-methyl-6-tert-butylphenol) and modephene. The benzene includes decahydro-4a-methyl-1-methylene-7-(1-methylethylidene)-(4aR, 8aS)-rel-naphthalene. Detailed information on the retention time, peak area, and m/z is shown in Supplementary Table S1.

**TABLE 1:** Analysis of volatile components in Hua-Feng-Dan by GC-MS.

Number	Component	Molecular formula/ion	Relative percentage (%)
1	Endo-Borneol	C_10_H_18_O	50.63
2	Isoborneol	C_10_H_18_O	35.87
3	2-NaphthaleneMethanol, decahydro-alpha,alpha,4a-triMethyl-8-Methylene-, (2R,4aR,8aS)-	C_15_H_26_O	1.52
4	(+)-2-Bornanone	C_10_H_16_O	1.17
5	Fenchol	C_10_H_18_O	1.12
6	Muscone	C_16_H_30_O	0.98
7	1-(Furan-2-yl)-4-methylpentan-1-one	C_10_H_14_O_2_	0.69
8	Naphthalene, decahydro-4a-methyl-1-methylene-7-(1-methylethenyl)-, (4aR,7R,8aS)-	C_15_H_24_	0.45
9	Bicyclo [2.2.1]heptan-2-ol, 1,7,7-trimethyl-, 2-acetate	C_12_H_20_O_2_	0.41
10	Agarospirol	C_15_H_26_O	0.34
11	[1,1′-Biphenyl]-4-carboxaldehyde	C_13_H_10_O	0.22
12	Caryophyllene oxide	C_15_H_24_O	0.17
13	9-Octadecenamide, (Z)-	C_18_H_35_NO	0.17
14	2-Naphthalenemethanol, 1,2,3,4,4a,5,6,7-octahydro-α,α,4a,8-tetramethyl-, (2S,4aR)-	C_15_H_26_O	0.15
15	Bicyclo [2.2.1]heptan-2-ol, 2,3,3-trimethyl-	C_10_H_18_O	0.12
16	Himachala-2,4-diene	C_15_H_24_	0.12
17	Cyclohexanemethanol, 4-ethenyl-.alpha.,.alpha.,4-trimethyl-3-(1-methylethenyl)-, (1R,3S,4S)-	C_15_H_26_O	0.12
18	1,3-Benzodioxole,4-Methoxy-6-(2-propen-1-yl)-	C_11_H_12_O_3_	0.11
19	Isobornyl propionate	C_13_H_22_O_2_	0.11
20	Berkheyaradulene	C_15_H_24_	0.08
21	Phenol, 2,2′-methylenebis [6-(1)	C_23_H_32_O_2_	0.07
22	Caryophyllene	C_15_H_24_	0.06
23	Guaia-9,11-diene	C_15_H_24_	0.06
24	(-)-Spathulenol	C_15_H_24_O	0.06
25	Phytol	C_20_H_40_O	0.06
26	Bornyl isovalerate	C_15_H_26_O_2_	0.05
27	1H-Cycloprop [e]azulen-7-ol, decahydro-1,1,7-trimethyl-4-methylene-, (1aR,4aR,7S,7aR,7bR)-ene-, (1aS,4aS,7S,7aS,7bS)-	C_15_H_24_O	0.05
28	Bornyl acetate	C_12_H_20_O_2_	0.04
29	Cyclohexanone,5-methyl-2-(1-methylethylidene)-	C_10_H_16_O	0.04
30	Modephene	C_15_H_24_	0.04
31	Bicyclo [7.2.0]undec-4-ene, 4,11,11-trimethyl-8-methylene-	C_15_H_24_	0.04
32	Hexadecanoic acid, ethyl ester	C_18_H_36_O_2_	0.04
33	gamma.-Elemene	C_15_H_24_	0.03
34	Cyclopenta [c]pentalene, decahydro-1,3a,5a-trimethyl-4-methylene-, (1R,3aR,5aS,8aR)-rel-	C_15_H_24_	0.03
35	Naphthalene, decahydro-4a-methyl-1-methylene-7-(1-methylethylidene)-, (4aR,8aS)-rel-	C_15_H_24_	0.03
36	Linalool	C_10_H_18_O	0.02
37	Isobornyl formate	C_11_H_18_O_2_	0.02
38	1,4,7,-Cycloundecatriene	93.05, 80.10, 121.05, 77.00, 91.00	0.02
39	2-Pentadecanone, 6,10,14-trimethyl-	C_18_H_36_O	0.02
40	Phthalic acid, cis-hex-3-enyl	149.00, 57.05, 150.05, 223.10, 104.00	0.02
41	Dibutyl phthalate	C_16_H_22_O_4_	0.02
42	Fenchone	C_10_H_16_O	0.01
43	Hexadecane	C_16_H_32_	0.01
44	4H-3a,7-Methanoazulene, 5,6,7,8-tetrahydro-1,4,9,9-tetramethyl-, (3aS,4R,7R)-	C_15_H_22_	0.01

The liposoluble constituents in the powder of Hua-Feng-Dan were extracted *via* solvent extraction. A total of 50 compounds were identified which were divided into 9 categories: alcohols (11, 20.49%), olefins (3, 10.21%), esters (5, 7.77%), amine (9, 5.10%), alkanes (6, 1.55%), acetate (2, 0.99%), ketones (3, 0.95%), benzene (2, 0.62), and phenols (1, 0.19%). Table 2 lists the 44 liposoluble components in Hua-Feng-Dan. The alcohols include endo-borneol, agarospirol, and alpha-bisabolol. The olefins include hexadecane, (3E, 5Z)-2,2,4,5,7,7-hexamethyl-3,5-octadiene, decahydro-4a-methyl-1-methylene-7-(1-methylethenyl)-, and (4aR,7R, 8aS)-naphthalene. The esters include 1,4-benzenedicarboxylic acid-bis(2-ethylhexyl) ester, iso-bornyl methacrylate, and bornyl acetate. The amines include (Z)-9-octadecenamide and 5-methoxy-2-methyl-benzenamine. The alkanes include hentriacontane, octadecane, and heneicosane. The acetates include n-hexadecanoic acid. The ketones include (+)-2-bornanone, muscone, and 4-hexanoylresorcinol. The phenols include 2,2′-methylene bis-(4-methyl-6-tert-butylphenol). The detailed information on the retention time, peak area, and m/z is shown in Supplementary Table S2.

**TABLE 2:** Analysis of liposoluble components in Hua-Feng-Dan by GC-MS.

Number	Component	Molecular formula/ion	Relative percentage (%)
1	Phosphonous dichloride, (1,7,7-trimethylbicyclo [2.2.1]hept-2-yl)-	137.05, 81.10, 95.10, 55.05, 69.10	19.12
2	Isobornyl caprate	136.05, 95.10, 137.05, 81.10, 93.10	12.33
3	Endo-Borneol	C_10_H_18_O	11.13
4	(3E,5Z)-2,2,4,5,7,7-Hexamethyl-3,5-octadiene	C_14_H_26_	10.12
5	Isoborneol	C_10_H_18_O	6.87
6	Trans, *trans*-2-ethylbicyclo [4.4	137.05, 81.10, 95.10, 79.10, 67.10	6.02
7	1,7,7-Trimethyl-,acetate, (1s-endo)-bicyclo [2.2.1]heptan-2-o	C_12_H_20_O_2_	5.13
8	Phosphoric acid, tribornyl ester	137.05, 81.10, 95.10, 79.10, 67.10	1.96
9	Benzenamine, 5-methoxy-2-methyl-	C_8_H_11_NO	1.63
10	9-Octadecenamide, (Z)-	C_18_H_35_NO	1.26
11	Hydrocinnamic acid, bornyl ester	137.05, 81.10, 95.10, 55.10, 69.10	0.94
12	1H-Pyrrole, 1-pentyl-	C_9_H_15_N	0.65
13	13-Docosenamide, (Z)-	C_22_H_43_NO	0.63
14	4-Hexanoylresorcinol	C_12_H_16_O_3_	0.62
15	beta.-Sitosterol	C_29_H_50_O	0.57
16	1,5,9-Undecatriene, 2,6,10-trimethyl-, (5Z)-	C_14_H_24_	0.54
17	Octadecane	C_18_H_38_	0.52
18	Hentriacontane	C_31_H_64_	0.52
19	Alpha.-Amyrin	C_30_H_50_O	0.52
20	2-Naphthalenemethanol, decahydro-α,α,4a-trimethyl-8-methylene-, (2R,4aR,8aS)-	C_15_H_26_O	0.5
21	Anthracene, 9-(2-propenyl)-	218.15, 203.15, 43.05, 107.10, 69.05	0.47
22	3-Methoxybenzylamine	C_8_H_11_NO	0.35
23	Heneicosane	C_21_H_44_	0.32
24	1,4-Benzenedicarboxylic acid, bis(2-ethylhexyl) ester	C_24_H_38_O_4_	0.31
25	Beta.-Amyrin	C_30_H_50_O	0.3
26	Iso-Bornyl methacrylate	C_14_H_22_O_2_	0.26
27	Muscone	C_16_H_30_O	0.25
28	Phenol, 3-(dimethylamino)-	C_8_H_11_NO	0.21
29	Phytol	C_20_H_40_O	0.19
30	Octadecanamide	C_18_H_37_NO	0.19
31	PHENOL,2,2′-METHYLENEBIS [6-(1)	C_23_H_32_O_2_	0.19
32	Alpha.-Bisabolol	C_15_H_26_O	0.15
33	Pyrene, hexadecahydro-	C_16_H_26_	0.15
34	Eicosane	C_20_H_42_	0.13
35	Hexadecanamide	C_16_H_33_NO	0.12
36	Bornyl acetate	C_12_H_20_O_2_	0.11
37	Agarospirol	C_15_H_26_O	0.11
38	Fenchol	C_10_H_18_O	0.1
39	(+)-2-Bornanone	C_10_H_16_O	0.08
40	Naphthalene, decahydro-4a-methyl-1-methylene-7-(1-methylethenyl)-, (4aR,7R,8aS)-	C_15_H_24_	0.08
41	Octadecanamide	C_18_H_34_O_2_	0.06
42	2-Naphthalenemethanol, 1,2,3,4,4a,5,6,7-octahydro-α,α,4a,8-tetramethyl-, (2R,4aR)-	C_15_H_26_O	0.05
43	n-Hexadecanoic acid	C_16_H_32_O_2_	0.05
44	1-(Furan-2-yl)-4-methylpentan-1-one	95.00, 110.10, 39.00, 41.00, 43.00	0.04
45	4-Methyl-3-(2-methylpropyl)-6-is	42.10, 100.10, 129.00, 185.10, 142.00	0.04
46	Hexadecane, 2,6,10,14-tetramethyl-	C_20_H_42_	0.04
47	Isobornyl laureate	136.05, 95.10, 93.05, 108.10, 57.00	0.03
48	(6R)-3,6β-Dimethyl-5,6,7,7aα-tetrahydrobenzofuran-2(4H)-one	C_10_H_14_O_2_	0.02
49	Pentadecane, 2,6,10-trimethyl-	C_18_H_38_	0.02
50	Hexadecane	C_16_H_32_	0.01

**FIGURE 2:**
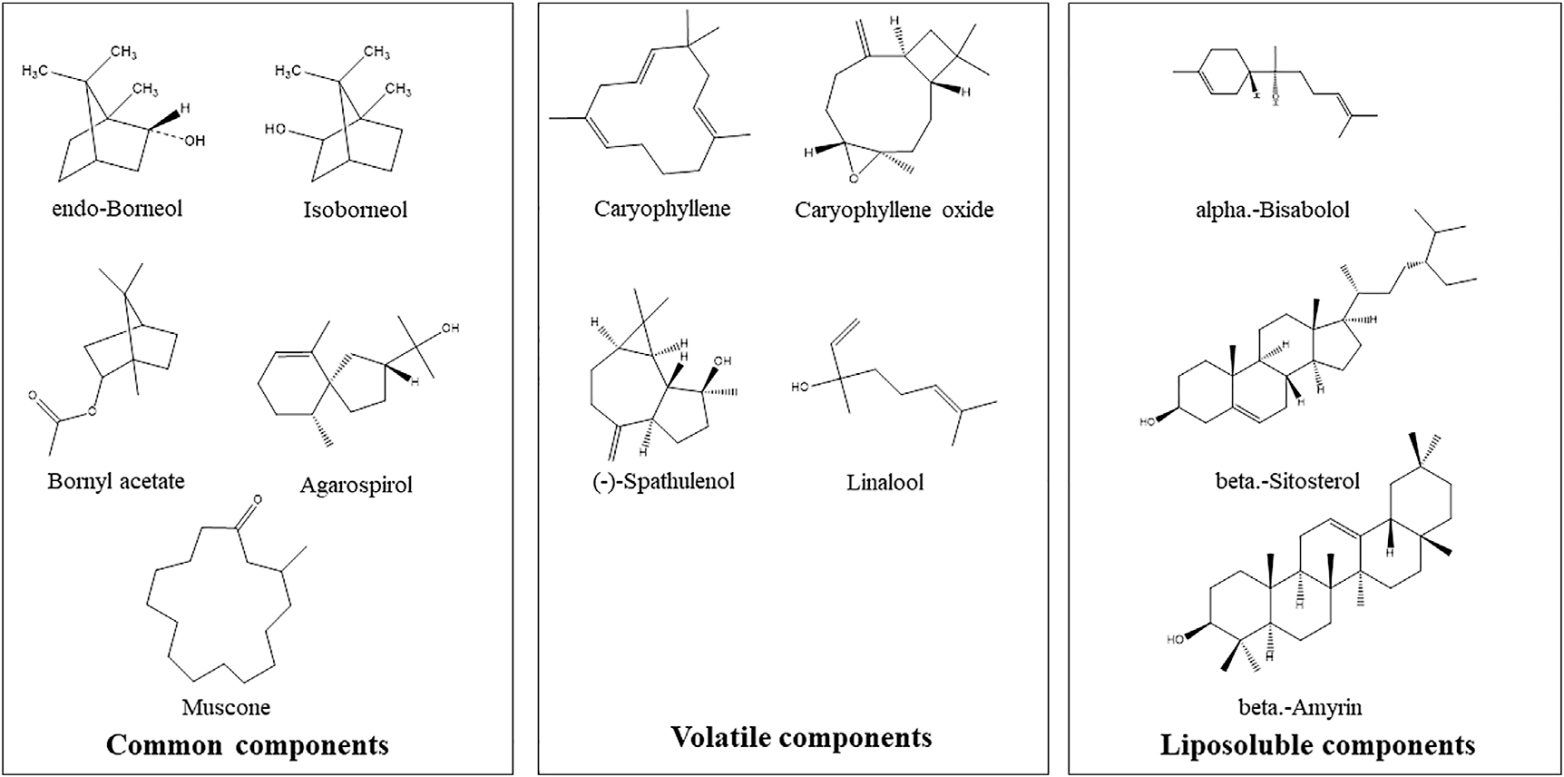
Examples of chemical structures of inferred characteristic compounds in Hua-Feng-Dan by GC-MS analysis.

The structures of some characteristic compounds are shown in Figure 2. The examples of identified compounds include endo-borneol, isoborneol, (-)-spathulenol, agarospirol, linalool, alpha-bisabolol, beta-amyrin, beta-sitosterol, muscone, bornyl acetate, caryophyllene oxide, and caryophyllene.

### 3.2 The Effect of Hua-Feng-Dan and Yaomu on Serum Enzyme Activities and Liver Pathology

Seven days after administration, the body weight of mice in each group increased slightly (the initial weights were 22.5 ± 0.51, 22.1 ± 1.16, 23.1 ± 1.28, and 23.3 ± 0.68 g for Control, Yaomu-0.1, Yaomu-0.3, and Hua-Feng-Dan groups, respectively; the final weights were 23.6 ± 1.08, 23.3 ±1.06, 23.9 ± 1.15, and 24.7 ± 0.66 g for Control, Yaomu-0.1, Yaomu-0.3, and Hua-Feng-Dan groups, respectively). All mice were in good health, the hair was shiny, the intake of diet and water was normal, and there were no other abnormal activities. Compared with the Control group (ALT, 14.5 ± 0.73 IU/l; AST, 19.0 ± 2.21 IU/l), there was no significant difference in serum ALT and AST of mice in the Yaomu-0.1 group (ALT, 13.9 ± 2.66 IU/l; AST, 23.2 ± 1.09 IU/L), Yaomu-0.3 group (ALT, 13.8 ± 3.06 IU/l; AST, 16.7 ± 3.62 IU/l), and Hua-Feng-Dan group (ALT, 22.3 ± 8.70 IU/L; AST, 20.0 ± 6.59 IU/L). These results confirmed our prior publications (Liu et al., 2018; Chen et al., 2020; Hu et al., 2020), indicating that Hua-Feng-Dan and Yaomu at the clinical dose did not cause hepatic toxicity.

**FIGURE 3:**
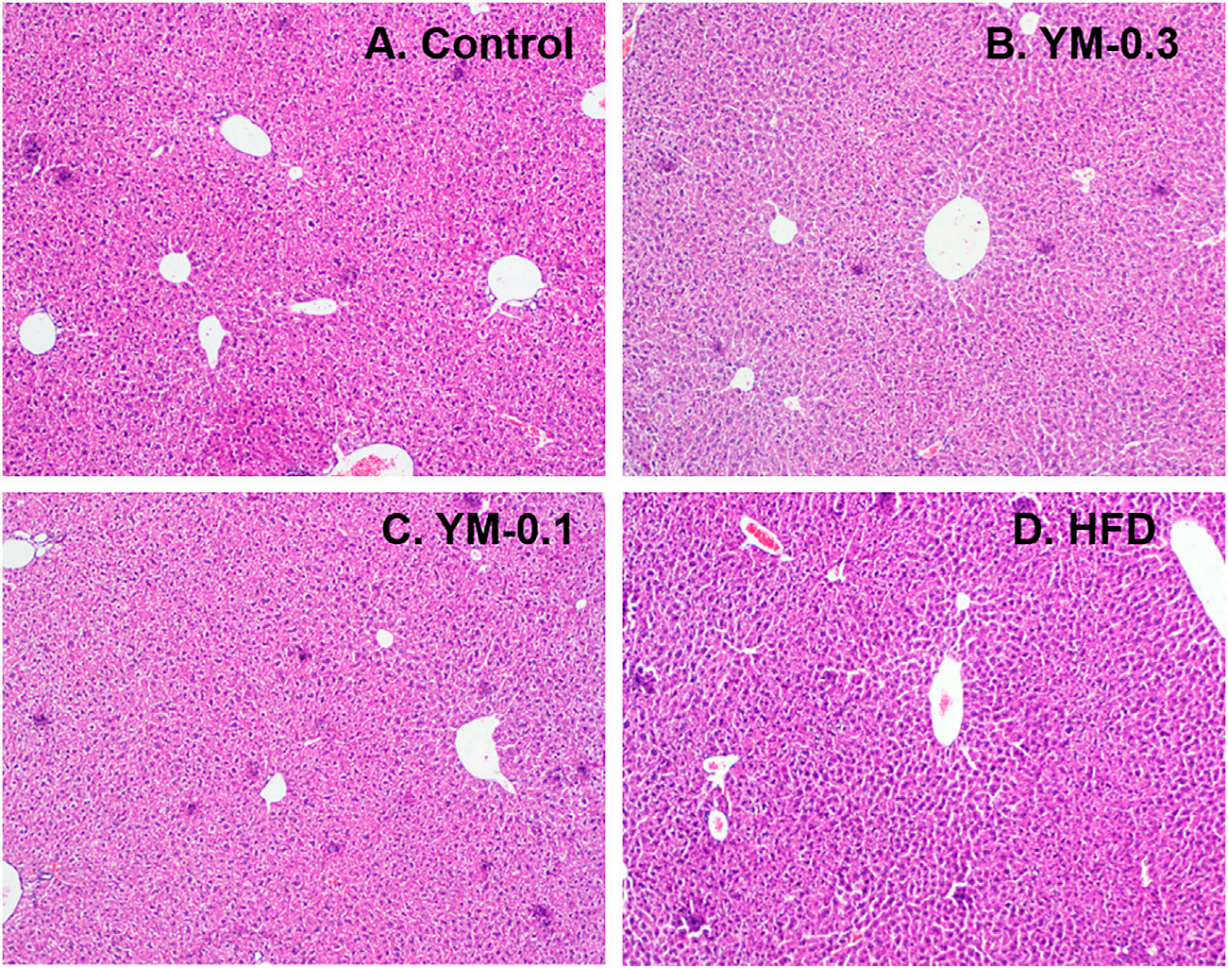
Representative photos of liver morphology. **(A)** Control. **(B)** YM-0.3. **(C)** YM-0.1. **(D)** HFD. Magnitude (×100).

The liver lobules of the mice in the Control group and treatment groups were clear and complete, without degeneration and necrosis of hepatocytes, and the liver cords were arranged radially around the central vein. No obvious pathological damage was observed in the liver after administration of different doses of Yaomu (YM) and Hua-Feng-Dan (HFD) to mice (Figure 3).

### 3.3 Gene Expression Pattern Overview

**FIGURE 4:**
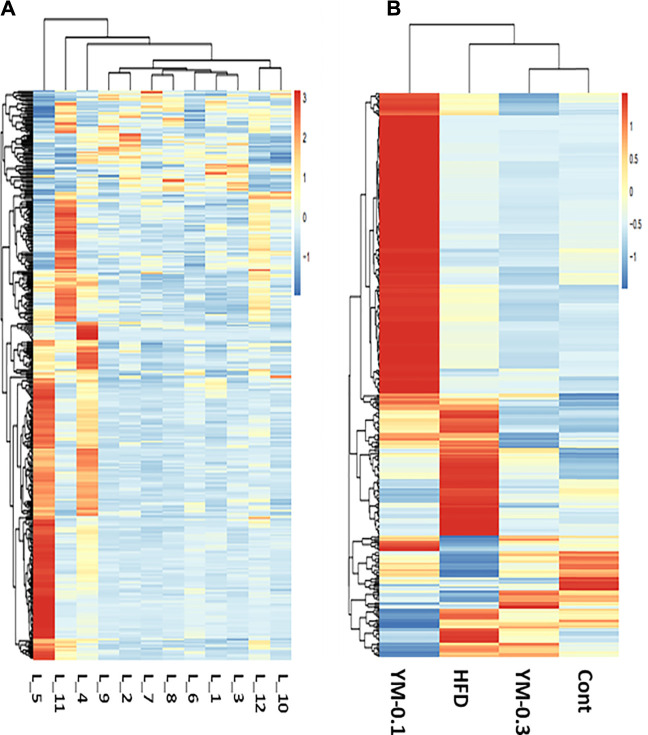
Overview of RNA sequencing results. **(A)** The clustering heatmap of gene expression from 12 individual samples. **(B)** The heatmap of gene expression from Cont, YM-0.3, HFD, and YM-0.1 groups. The color brightness is associated with differences in multiples.

RNA-Seq-generated FPKM were transformed to raw gene counts after initial quality control analysis (Zhang et al., 2021). The results (∼22,500/sample) were subjected to cluster analysis. The clustering heatmaps of gene expressions from 12 individual samples (Figure 4A) and 4 groups (Figure 4B) are shown. Red represents the upregulation; blue represents the downregulation. The color brightness is associated with differences. Figure 4A shows the heatmap of 12 individual samples. Some of the three samples in each group showed similar patterns (L-1, L-2, and L-3, Control group; L-7, L-8, and L-9, YM-0.3 group), while in the YM-0.1 group, L-5 was somewhat different from L-4 and quite different from L6. In the HFD group, L-11 was separated from L-10 and L-12. Figure 4B shows the heatmap of 4 groups; YM-0.3 was slightly different from Control, but YM-0.1 and HFD had clearly different patterns from Control.

### 3.4 GO and KEGG Pathway Enrichment Analysis

**FIGURE 5:**
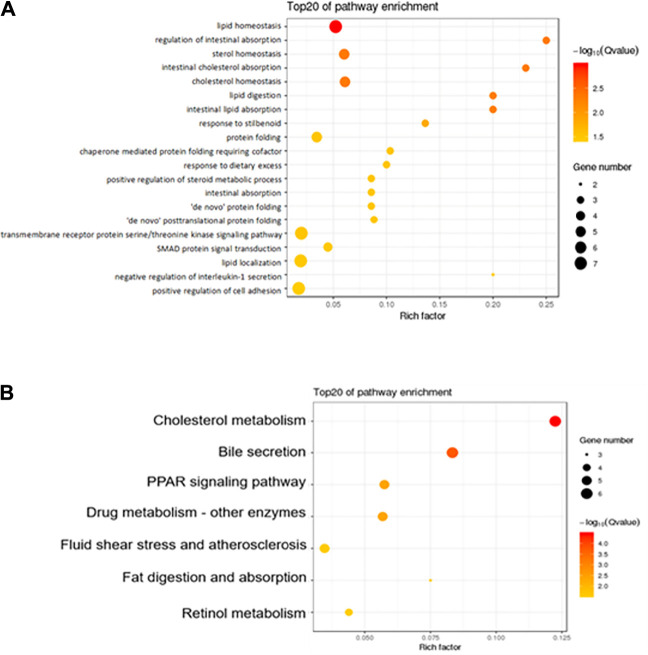
Functional enrichment of the differentially expressed genes. **(A)** GO analysis shows the biological processes involved in DEGs. **(B)** KEGG shows the signaling pathway involved in DEGs. The size of the dot in the figure represents the number of DEGs that can be annotated in the functional pathway, and the shade of the color represents the significant degree of enrichment of the functional pathway.

The GO database standardizes the description of DEGs in terms of function, participating biological pathways, and cell location. KEGG is a database of the metabolic pathways of gene products in cells and the functions of these gene products (Zhang et al., 2021). Here we focus on the comparison between the Hua-Feng-Dan and Control groups. Figure 5 summarizes the top 20 enriched by GO and the top 7 enriched by the KEGG pathway. GO enrichment analysis shows that the biological process of DEGs was mainly involved in lipid metabolism, sterol homeostasis, cholesterol homeostasis, intestinal absorption, protein folding, and cell adhesion (Figure 5A). KEGG enrichment analysis shows that pathways of DEGs were mainly involved in cholesterol metabolism, bile secretion, PPAR signaling pathway, drug metabolism, fat digestion and absorption, and retinol metabolism (Figure 5B).

### 3.5 Differentially Expressed Gene Analysis

**FIGURE 6:**
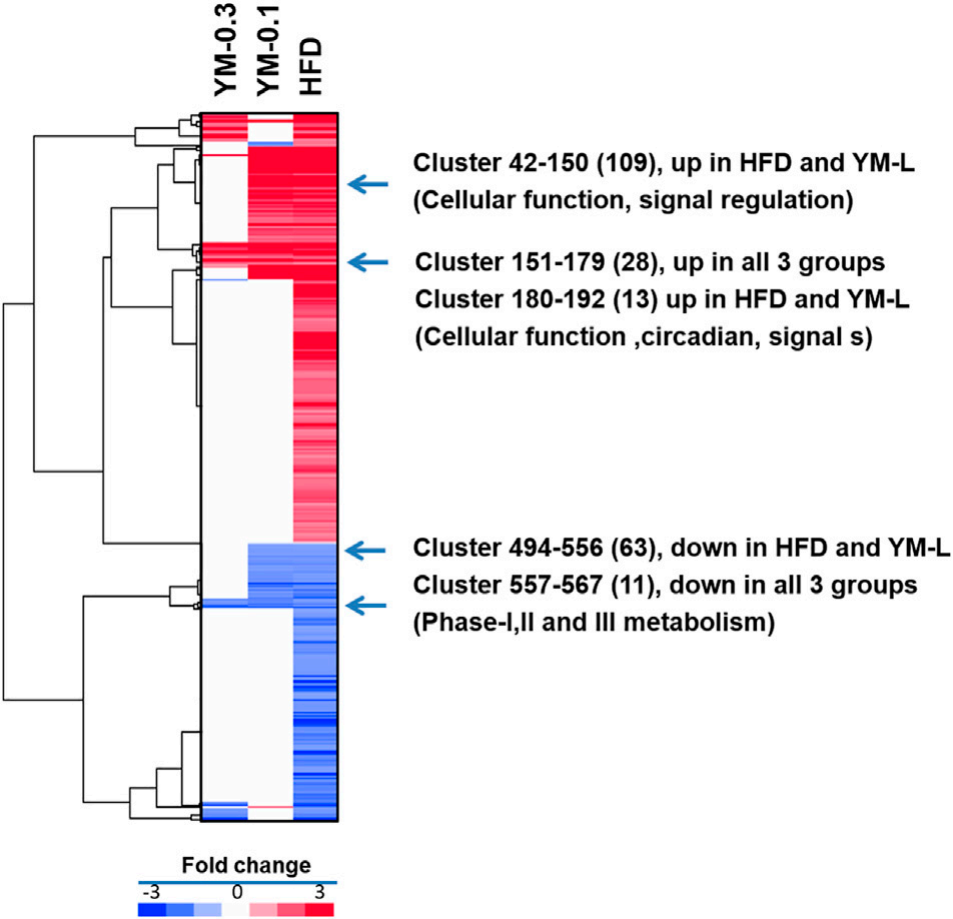
Two-dimensional clustering analysis of DEGs compared to Control and based on the HFD group. YM-0.3 group (First column 92 DEGs). The YM-0.1 group had 235 DEGs (second column), and HFD had 806 DEGs (third column).

DEGs were analyzed *via* the DESeq2 method compared to Controls at *p* < 0.05. Using the complete two-dimensional clustering, the DEGs were clustered and input into TreeView to visualize the differences between groups (red indicates upregulation and blue indicates downregulation) (Zhang et al., 2021). From Figure 6, compared to the HFD group (806 DEGs), the YM-0.1 group had 235 DEGs and the YM-0.3 group had 92 DEGs. Four clusters were selected for annotation: two upregulated clusters: in lines 42-150, 109 genes were increased in the HFD and YM-0.1 groups only, mainly involved in cellular function and signal regulation; in lines 151-179, 28 genes involved in cellular function, circadian, and signal transduction were increased in all three groups. Two downregulated clusters: in lines 494-556, 63 genes were decreased in the HFD and YM-0.1 groups only, while in lines 557-567, 11 genes were decreased in all three groups. These genes are involved in Phase-I, II, and III metabolism and immunomodulation. The clustered DEGs with the annotation for gene names are shown in 3-4-Supplementary Table S2-DEGs.

### 3.6 qPCR Analysis of Selected DEGs

**FIGURE 7:**
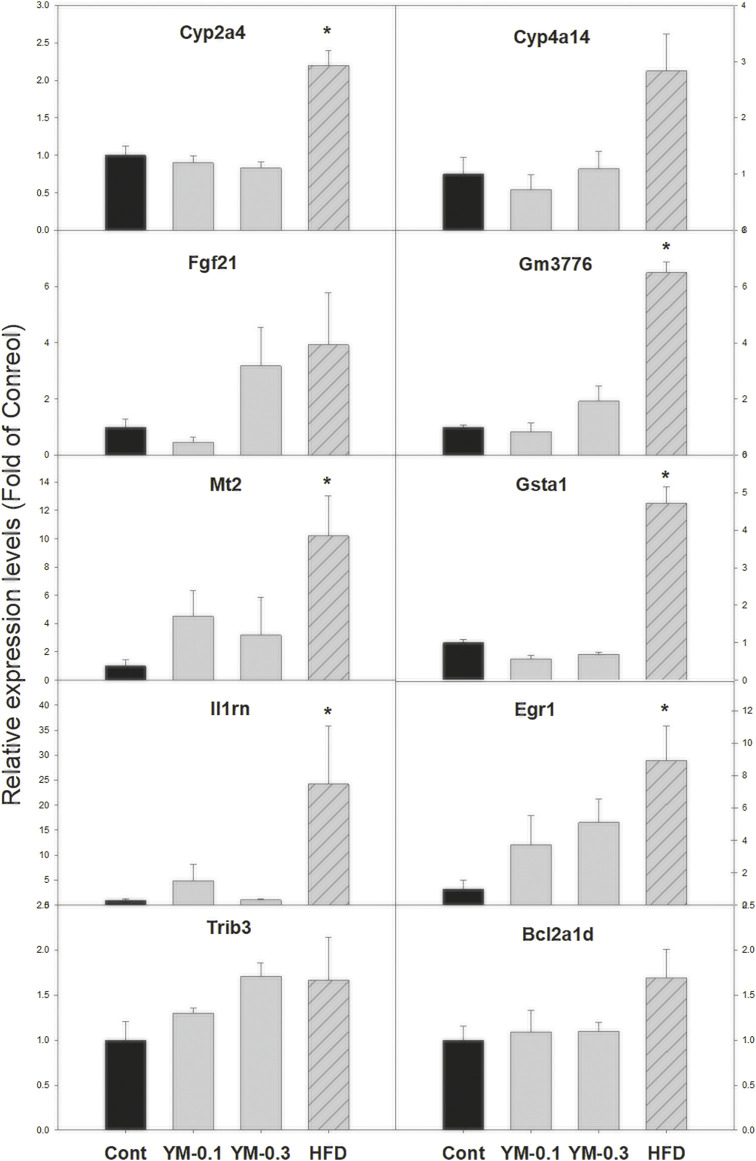
qPCR analysis of 10 selected DEGs. Total RNA was transcribed, and qPCR was performed with the specific primers listed in Supplementary Table S1. Data are mean ± SEM of Control (*n* = 5), YM-0.1 (*n* = 5), YM-0.3 (*n* = 7), and HFD (*n* = 5). Data were subjected to Kruskal–Wallis one-way ANOVA, followed by Dunn’s multiple-comparison test. *Significantly different from Control *p* < 0.05.

Ten selected DEGs were further analyzed *via* qPCR (Figure 7). The expression of Cyp2a4 was increased 2.2-fold by Hua-Feng-Dan (HFD) but was unaffected by YM-0.1 and YM-0.3; the expression of Cyp4a14 was increased 2.3-fold by HFD, but YM-0.1 and 0.3 did not affect its expression. The expression of Fgf21 was increased by HFD (3.7-fold), although not reaching significance; the expression of Gm3776 was increased 6.5-fold by HFD; the expression of Mt2 (10-fold) and Gsta1 (4.7-fold), Il1rn (24-fold), and Egr1 (8.9-fold) was markedly increased by HFD. The expression of Trib3 (1.7-fold) and Bcl2a1d (1.7-fold) tended to increase but was not significant. The two doses of YM had little effects on the expression of these genes.

### 3.7 Ingenuity Pathways Analysis of DEGs

**FIGURE 8:**
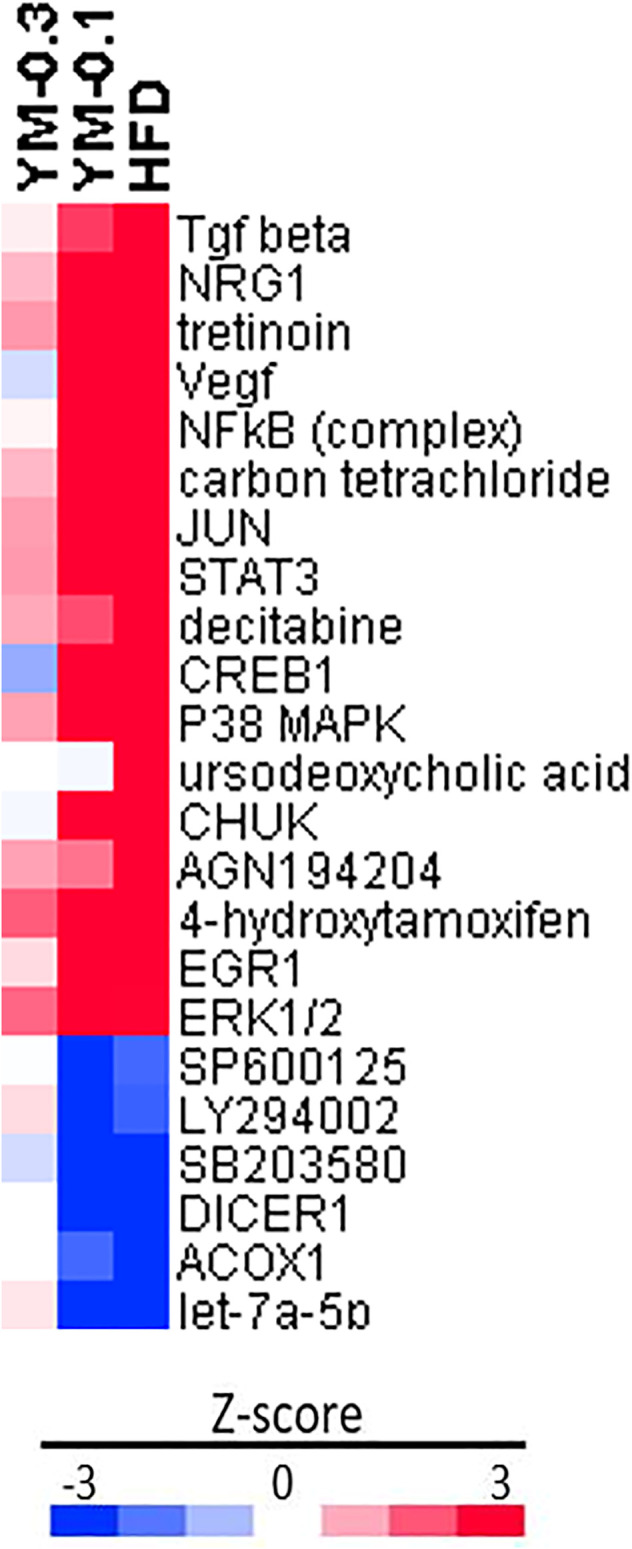
Ingenuity pathway analysis (IPA) of upstream regulators based on HFD in Z-score. Red indicates the upregulation, and blue indicates the downregulation.

IPA upstream regulator analysis (Figure 8) was used to identify the upstream regulators that may be responsible for gene expression changes observed in the study. IPA predicts which upstream regulators are activated or inhibited to explain the upregulated and downregulated genes observed. The top 25 upstream regulators were selected, including 18 increased by HFD with molecules (Tgf beta; NRG1; Vegf; NFκB (complex); JUN; STAT3; CREB1; P38 MAPK; CHUK; EGR1; ERK1/2; and EP300) or with chemicals (Tretinoin; carbon tetrachloride; decitabine; ursodeoxycholic acid; AGN194204; and 4-hydroxytamoxifen). The 7 downregulated regulators were molecules (Let-7a-5p, ACOX1, and DICER1) and chemicals (MEK1/2 inhibitor U0126; p38 MAPK inhibitor SB203580; JNK inhibitor SP600125; and PIK3 inhibitor LY294002). All of these upstream regulators point toward the activation of MAPK signaling pathways and induced adaptive responses. The downregulation of inhibitors for MAPK and PIK3 signaling pathways further supported induced adaptive responses. YM-0.1 produced similar upstream regulator alternations in the similar direction, except for ursodeoxycholic acid, while the high dose of YM-0.3 produced weak alterations in these regulators, with 3 in opposite directions (different color).

### 3.8 Illumina BaseSpace Correlation Engine Analysis of DEGs

**FIGURE 9:**
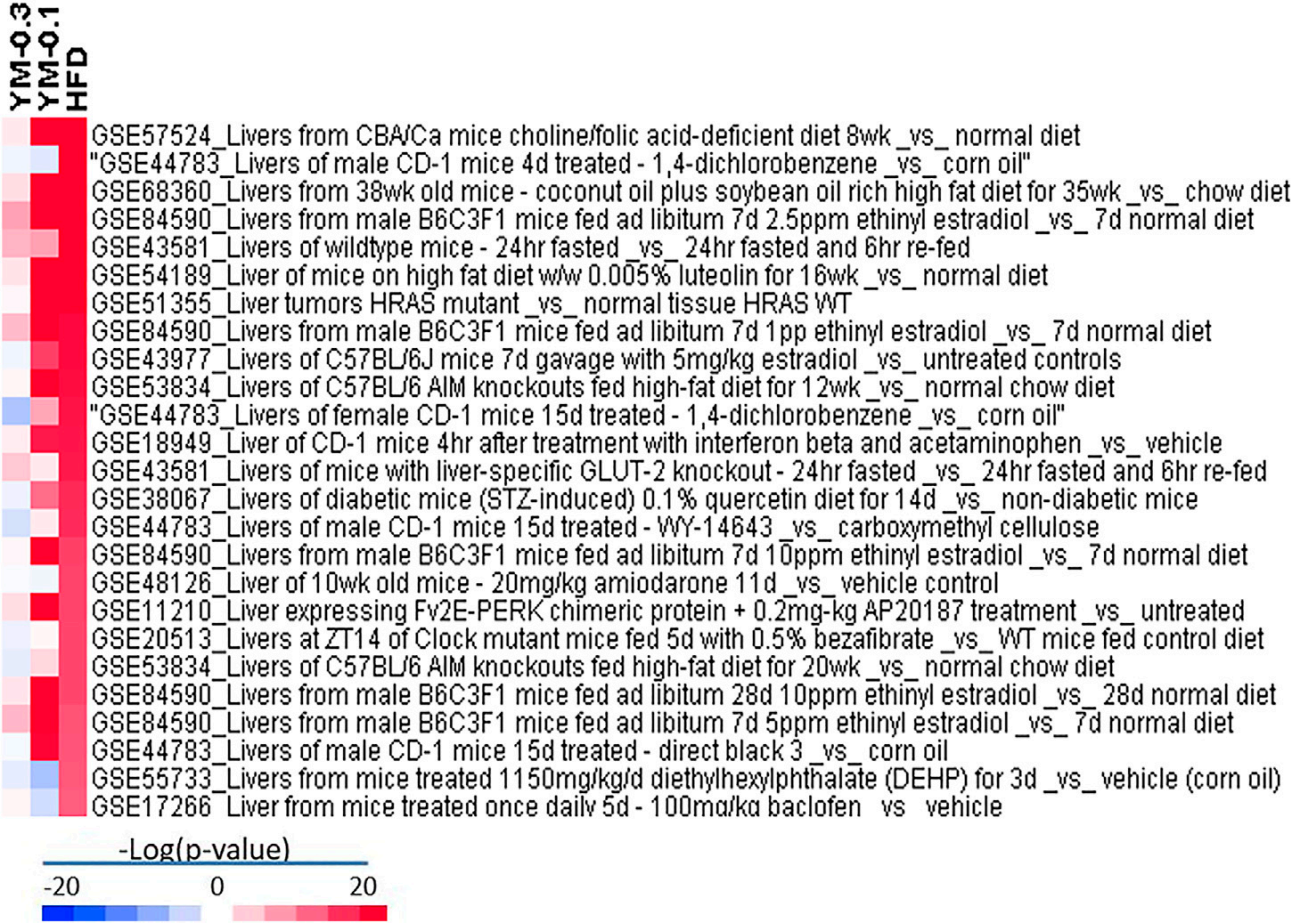
BaseSpace Correlation Engine (BSCE) analysis of DEGs based on the HFD group in –log (p-value). Red indicates the upregulation, and blue indicates the downregulation. The GSE numbers in the Gene Expression Omnibus (GEO) database were included.

All the biosets were imported into Illumina BSCE for curated studies, filtered by mice, RNA expression, and treatment vs. control, and the curated files were exported and the −log (p-value)s were calculated. VLOOKUP function was used for comparisons with the GEO database. There were very high correlations between HFD-induced DEGs, and 25 GSE databases with −log (p-value) from 12 to 26.9 were presented (Figure 9). The deeper the red color, the higher the −log (p-value). The value >4 is considered significant (Corton et al., 2019; Jackson et al., 2020). YM-0.1 moderately correlated with 18 GSE databases in the same direction but to a lesser extent, while YM-0.3 only weakly correlated with 9 databases and negatively correlated with 3 databases (blue color). Thus, Hua-Feng-Dan at the hepatoprotective dose mimics chemical-induced adaptive responses, while YM-0.1 could mimic some effects of HFD, but YM-0.3 has little effect, suggesting that the use of Yaomu at appropriate doses is important to induce adaptive responses.

## 4 Discussion

This study used GC-MS, and the NIST library primarily identified 44 volatile and 50 liposoluble components in Hua-Feng-Dan. Hua-Feng-Dan and its “Guide Drug” Yaomu at hepatoprotective doses did not produce damage to the liver but produced gene expression changes. RNA-Seq revealed 806 DEGs in Hua-Feng-Dan-treated livers compared to controls, and a low dose of Yaomu produced 235 DEGs in the same direction. GO and KEGG analyses revealed that Hua-Feng-Dan affected metabolism and signaling pathways. IPA upstream analysis pointed toward the activation of MAPK signaling pathways and induced adaptive responses by Hua-Feng-Dan. qPCR verified 10 selected DEGs. Hua-Feng-Dan-induced gene expression changes were highly correlated with the GEO database of chemical-induced adaptive responses in mouse liver.

The patented Chinese medicine Hua-Feng-Dan contains 8 kinds of medicinal herbs, 3 kinds of animal-based products, and 3 kinds of minerals (Liu et al., 2018). The present study is the first to profile volatile and liposoluble components in Hua-Feng-Dan, using GC-MS technology combined with NIST library analysis (Sun et al., 2018; Xu et al., 2020; Zhou et al., 2020) to primarily identify and quantify volatile and liposoluble components in Hua-Feng-Dan (Figures 1, 2). Although the volatile and liposoluble components in Hua-Feng-Dan were identified by GC-MS, and the quality standard level of Hua-Feng-Dan was improved, the compounds identified were speculated after their mass spectra were retrieved from the NIST14 database and were not confirmed by reference substances. This conjecture may not be entirely accurate, and the identifications reported for this research are Level 2 according to the MSI which stands for putatively annotated compounds (Sumner et al., 2007). Therefore, the relevant components need to be further verified. The composition of Hua-Feng-Dan is complex, and the chemical components such as phenols, flavonoids, terpenoids, alkaloids, and glycosides can be further analyzed by LC-MS to confirm the target compounds, and the compounds can be further quantitatively analyzed.

Table 1 lists >1% volatile components. Endo-borneol was the major ingredient accounting for 50.63%. Endo-borneol is the major component in *Stachys viticina* Boiss, and the oil extracts exhibited antioxidant and metabolic regulation activities (Jaradat and Al-Maharik, 2019). Endo-borneol is also the major component in *Artemisia gmelinii*, and the oil extracts exhibited antioxidant, antidiabetic, and anticholinesterase activities (Xu et al., 2021). Table 2 lists >1% liposoluble components. For example, isoborneol had a relative percentage of 6.87%. Bornerol is used in Chinese medicines for thousands of years and had profound effects on liver metabolic enzymes CYP2B (Chen et al., 2017) and CYP3A (Zhang et al., 2013) and hepatic drug transporters (Chen et al., 2019). Isobornyl caprate had a relative percentage of 12.33%. Sodium caprate could synergistically increase the hypoglycemic effects of berberine through the AMPK pathways (Zhang et al., 2012b). The network pharmacology-predicted anti-stroke ingredient beta-sitosterol (Yang et al., 2020) is also among the identified molecules (Figure 2). In addition to cinnabar (Hg) and realgar (As), other trace elements such as Cu, Cd, and Pb can also be determined in Hua-Feng-Dan by ICP-MS (Zhao and Zhang, 2019).

Through literature review, the medicinal materials from which the identified compounds may be derived were inferred, and it was found that alkaloids, organic acids, volatile oils, and steroids, such as beta-sitosterol, may be derived from *Pinellia ternata* (Wang and Wang, 2020); acetic acid, 9-octadecenamide, (Z)-, phytol, 2-pentadecanone, 6,10,14-trimethyl-, octadecanamide, n-hexanoic acid and other phenolic and organic acids, nitrogen, sugars, and volatile components may be derived from *Gastrodia elata* Bl (Han et al., 2018; Jin et al., 2021). Volatile oil components such as caryophyllene oxide may be derived from *Nepeta tenuifolia* Benth. (Cheng et al., 2021); other volatile oil compounds such as agarospirol and caryophyllene oxide may be derived from *Atractylodes lancea* (Thumb.) DC (Pang et al., 2020). Volatile oil compounds such as caryophyllene oxide, linalool, and 1-hexadecane may be derived from *Perilla frutescens* (L.) Britt. (Li et al., 2018); alpha-amyrin, beta-amyrin, and other sesquiterpenes, monoterpenes, and lignans may be derived from *Santalum album* L. (Zhang et al., 2020). It is helpful to further clarify the medicinal material basis of Hua-Feng-Dan to judge the medicinal material source of identified compounds. However, these components could act together in an integrated manner, not as individual pure compound as seen in Western medicines. Therefore, the use of the entire recipe though oral administration to define pharmacological effects is becoming the next goal of this study.

We have successfully used RNA-Seq to dissect the protective mechanism of *Dendrobium nobile* alkaloids to protect against CCl_4_-induced liver injury (Zhang et al., 2021) and demonstrated that the adaptation is the major mechanism of hepatoprotection for oleanolic acid (Liu et al., 2019), similar to the “program the liver” concept (Klaassen, 2014). The hepatoprotection potential of Hua-Feng-Dan promoted us to use the same strategy to examine whether adaptive mechanisms exist for this traditional Chinese medicine.

In the present study, Hua-Feng-Dan and Yaomu at hepatoprotective doses did not produce liver injury as evidenced by serum enzyme activities and histopathology, consistent with prior observations (Yan et al., 2011; Peng et al., 2012; Liu et al., 2018). This is an important pre-requirement for RNA-Seq experiments. GO enrichment showed top 20 enrichments Hua-Feng-Dan could modulate. The top 3 KEGG pathways include cholesterol metabolism, bile secretion, and PPAR signaling. Cholesterol homeostasis is important for biological functions and bile acid synthesis and PPAR signaling pathway, and drug metabolism genes are important for liver functions (Boyer, 2013; Luo et al., 2020). Hua-Feng-Dan effects on these pathways imply its ability to “program the liver” to induce adaptive responses.

Two-dimensional clustering analysis of DEGs showed that Hua-Feng-Dan upregulated genes of cellular function, signal regulation, and circadian and signal regulation and reduced the expression of genes related to Phase-I, II, and III xenobiotic metabolism, endogenous metabolism, disease traits, and immune modulation, all pointing toward adaptive responses. For example, induction of Cyp2a4 plays roles in circadian regulation and liver detoxification (Zhao et al., 2019); induction of Cyp4a14 may contribute to increased resistance to oxidative stress (Sobocanec et al., 2010); induction of Fgf21 plays a role in nonalcoholic fatty liver disease (NAFLD) in humans and limits hepatotoxicity in mice (Desai et al., 2017); Gm3776 is a CAR-target gene in liver of mice as an adaptive response to xenobiotics (Cui and Klaassen, 2016); metallothioneins (Mt2) are sulfhydryl-rich small proteins for heavy metal detoxification and free radical scavenging (Klaassen et al., 1999); glutathione S-transferase A1 (Gsta1) is a phase II enzyme playing adaptive against toxic stimuli (Cheng et al., 2014); interleukin-1 receptor antagonist (Il1rn) is a member of the interleukin 1 cytokine family, inhibits the activities of interleukin 1α (IL-1α) and interleukin 1β (IL1-β), and prevents IL-1β overexpression during inflammatory responses (Meier et al., 2019); and early growth response 1 (Egr1) is an important acute-phase adaptive protein (Magee and Zhang, 2017). Upregulation of these genes points toward the adaptive machinery which is activated by Hua-Feng-Dan in an attempt to program the liver to produce hepatoprotective effects.

An IPA analysis of upstream regulatory factors clearly indicated the activation of the MAPK signaling pathways. For example, increased Tgf beta and Neuregulin 1 (NRG1) could regulate cell fate decisions during embryonic development, tissue homeostasis, and regeneration (David and Massagué, 2018; Zhang et al., 2018). Vascular endothelial growth factor (Vegf) is a subfamily of growth factors and could mediate liver injury (Oberkofler et al., 2014). Nuclear factor kappa B (NF-κB) is a protein complex that controls the transcription of DNA, cytokine production, and cell survival and plays an important role in adaptive immune response (Rahman and McFadden, 2011). These upstream molecules imply that the adaptive machinery is activated. On the other hand, the downregulation of the PI3K inhibitor LY294002 (Mazumder et al., 2019), p38 MAPK inhibitor SB203580 (Yan et al., 2016), and MEK1/2 inhibitor U0126 (Wang et al., 2018) further facilitated the activation of these adaptive responses.

The BSCE correlation analyses further strengthen Hua-Feng-Dan-induced adaptive responses. For example, mice treated with CD-1 for 4 days with 1,4-dichlorobenzene (GSE 44783) (Eichner et al., 2013) had a −log (p-value) of 26.9 for Hua-Feng-Dan; mice fed with a soybean oil-rich high-fat diet (GSE68360) (Deol et al., 2015) had a -log (p-value) 26.4 for Hua-Feng-Dan; and mice fed with luteolin which could reprogram the liver to ameliorate deleterious effects of diet-induced obesity (GSE54189) (Kwon et al., 2015) had a −log (p-value) of 20.7 with Hua-Feng-Dan. The effects of unfolded proteins in the endoplasmic reticulum (ER stress) on glucose intolerance in eIF2 (alphaP) transgenic mouse gene profiling (GSE11210) (Oyadomari et al., 2008) had a −log (p-value) of 14.5 with Hua-Feng-Dan. Clock mutant mice fed with bezafibrate, a PPARα activator, which affected the circadian clock, lipid metabolism, and liver gene expression (GSE20512) (Oishi et al., 2010), had a −log (p-value) of 14.2 with Hua-Feng-Dan. Thus, Hua-Feng-Dan at the hepatoprotective dose evoked hepatic gene expression changes toward adaptive responses. These changes could be beneficial or detrimental, similar to the ability of oleanolic acid to program the liver to protect against various hepatotoxicants, but it is hepatotoxic at high doses (Liu et al., 2019). Caution should be taken when using Hua-Feng-Dan at the high dose and for long periods.

## 5 Conclusion

This study preliminarily profiled volatile and liposoluble components in Hua-Feng-Dan *via* GC-MS and analyzed hepatic transcriptome changes after Hua-Feng-Dan and its “Guide Drug” Yaomu. Hua-Feng-Dan induced adaptive responses without causing apparent toxicity to program the liver. A low dose of Yaomu produced similar but fewer changes compared to Hua-Feng-Dan, while the high dose of Yaomu had little effect, suggesting that an appropriate dose of Yaomu is important. The effects of Hua-Feng-Dan on the liver transcriptome changes were highly correlated with chemical-induced adaptive responses in the livers of mice in the GEO database. Thus, Hua-Feng-Dan could “program the liver” to produce adaptive responses, which could be the pharmacological basis for it to produce beneficial effects.

## Data Availability

The original contributions presented in the study are included in the article/Supplementary Material; further inquiries can be directed to the corresponding authors.

## Abbreviations

BSCE, Illumina BaseSpace Correlation Engine; DEGs, differentially expressed genes; GC-MS, gas chromatography–mass spectrometry; GO, gene ontology; HFD, Hua-Feng-Dan; IPA, ingenuity pathway analysis; KEGG, Kyoto Encyclopedia of Genes and Genomes; NIST, the National Institute of Standards and Technology; PCA, principle component analysis; YM, Yaomu, the “Guide drug” of Hua-Feng-Dan.
